# Do working length and proximal screw density influence the velocity of callus formation in distal tibia fractures treated with a medial bridge plate?

**DOI:** 10.1007/s00590-023-03697-6

**Published:** 2023-08-29

**Authors:** Antonio Gilli, Stefano Ghirardelli, Pierrenzo Pozzi, Georgios Touloupakis, Matteo Messori, Emmanouil Theodorakis, Guido Antonini

**Affiliations:** 1 Department of Orthopedic and Traumatology, San Carlo Borromeo Hospital, Milan, Italy; 2 University of Milan, Milan, Italy; 3 Women’s College Hospital, Orthopaedic Sports Medicine, University of Toronto, Toronto, Canada

**Keywords:** Working length, Screw density, Tibial fractures, Bridge plate, Bone healing

## Abstract

**Introduction:**

Aim of our study was to evaluate the influence of working length and screw density on callus formation in distal tibial fractures fixed with a medial bridge plate.

**Materials and methods:**

42 distal tibia fractures treated with a bridge plate were analyzed. Minimum follow-up was 12 months. mRUST score (modified Radiographic Union Scale for Tibial fractures) was used to assess callus formation. Working length and screw density were  measured from post-operative radiographs.

**Results:**

39 (92.9%) fractures healed uneventfully. 32 (76.19%) patients showed signs of early callus formation 3 months post-surgery. In these patients a lower screw density was used compared to patients who didn’t show early callus (33.4 vs. 26.6; *p* = 0.04). No differences was noticed in working length.

**Conclusion:**

Bridge plate osteosynthesis is a good treatment option in distal tibia fractures. In our series increasing the working length was not associated with a faster callus formation in distal tibia fractures. Conversely, a lower screw density proximally to the fracture site was associated to a faster callus growth.

## Introduction

Distal tibial fractures account for 3–10% of all tibial fractures or 1% of lower extremities fractures [1]. As these fractures are often caused by high-energy trauma, the association with bone comminution and extensile soft tissue damage is very common. Comminution at the fracture site makes anatomic reduction unfeasible. Aiming for a secondary bone healing through a bridge plate is the treatment associated with higher rates of success [2].

Controversy exists on how to recreate the most biomechanically effective environment for bridge plate osteosynthesis. Many studies have investigated how plate design, plate length and screw configuration can influence fixation stability and promote fracture healing, often with unclear results.

One of the most important parameters regulating plate-screws stiffness and construct stability is working length which is defined as the distance between the two innermost screws on either side of the fracture. Larger working length makes fixation more flexible, in an effort to reduce strain and stress on the plate [3].

Another parameter that has been investigated in previous studies is screw density. This parameter influences system rigidity. It has been suggested empirically that half of the holes in the plate should be occupied by screws [4].

Many biomechanical studies on locking plates focused on diaphyseal fractures of the femur, where a positive association between working length and fracture healing was demonstrated. Few studies have investigated these associations in the treatment of distal tibial fractures. The aim of this study is to analyze the influence of working length and screw density on distal tibia fractures fixed with a medial bridge plate. Specifically, we investigated how these parameters affect the velocity of callus formation at 3 months and the fracture healing at 1 year.

## Material and methods

This is a retrospective analysis of a consecutive series of patients treated for distal tibia fractures at authors’ institution from January 2014 to December 2019. All fractures treated with a medial bridge plate were included. The same plate (LCP Metaphyseal Plate for distal medial tibia 3.5–4.5, Depuy Synthes, Warsaw, IN) was used in all patients but in different sizes. In all patients only locking screws were used proximally to fracture site. Patients were excluded when non-locking or hybrid fixation was used proximally. Patients treated with a different plate were excluded. Distal tibia fractures that were fixed using more than one bridge plate were excluded. Fractures treated using the aforementioned Distal Tibia LCP plus a fibular plate were included. Fractures treated with intramedullary nail, lag screw or compression plate were also excluded. Patients’ demographics were recorded. We excluded patients under 18 year and over 85 years, patients with neuromuscular diseases or diseases affecting bone metabolism, heavy smokers (2 packs/day) and patients with previous fractures in the affected limb. Open fractures were also excluded.

Two senior trauma surgeons (> 10 years of experience) classified the fractures according to the AO/OTA classification based on preoperative X-rays and CT scans.

### Surgical procedure

All procedures were performed by the two aforementioned fellowship-trained trauma surgeons. Informed consent was obtained from all patients prior to surgery. In high energy fractures with severe tissue swelling a two-stage treatment was performed. First an external fixator spanning the ankle joint was applied and then, at oedema subsidence, definitive fixation was performed 7–15 days after trauma. A single shot of cefazolin or clindamycin was administered. Under general or spinal anesthesia, patients were positioned supine on a radiolucent table. No tourniquet was used during the surgical procedure. Whenever possible tibial fracture was reduced percutaneously or with a surgical approach centered on the fracture site. After adequate reduction, all tibia fractures were fixed with a medial bridge plate. Percutaneous osteosynthesis was preferred whenever technically feasible. If a fibula fracture was associated, it was fixed based on surgeon preference and experience. The patients began ankle mobilization on postoperative day 1. Toe-touch weight bearing was allowed from the second post-operative week. From the forth to the tenth post-operative week partial weight bearing (20 kg) was prescribed and after that weight bearing as tolerated was allowed.

### Biomechanical parameters evaluation

**Fig. 1:**
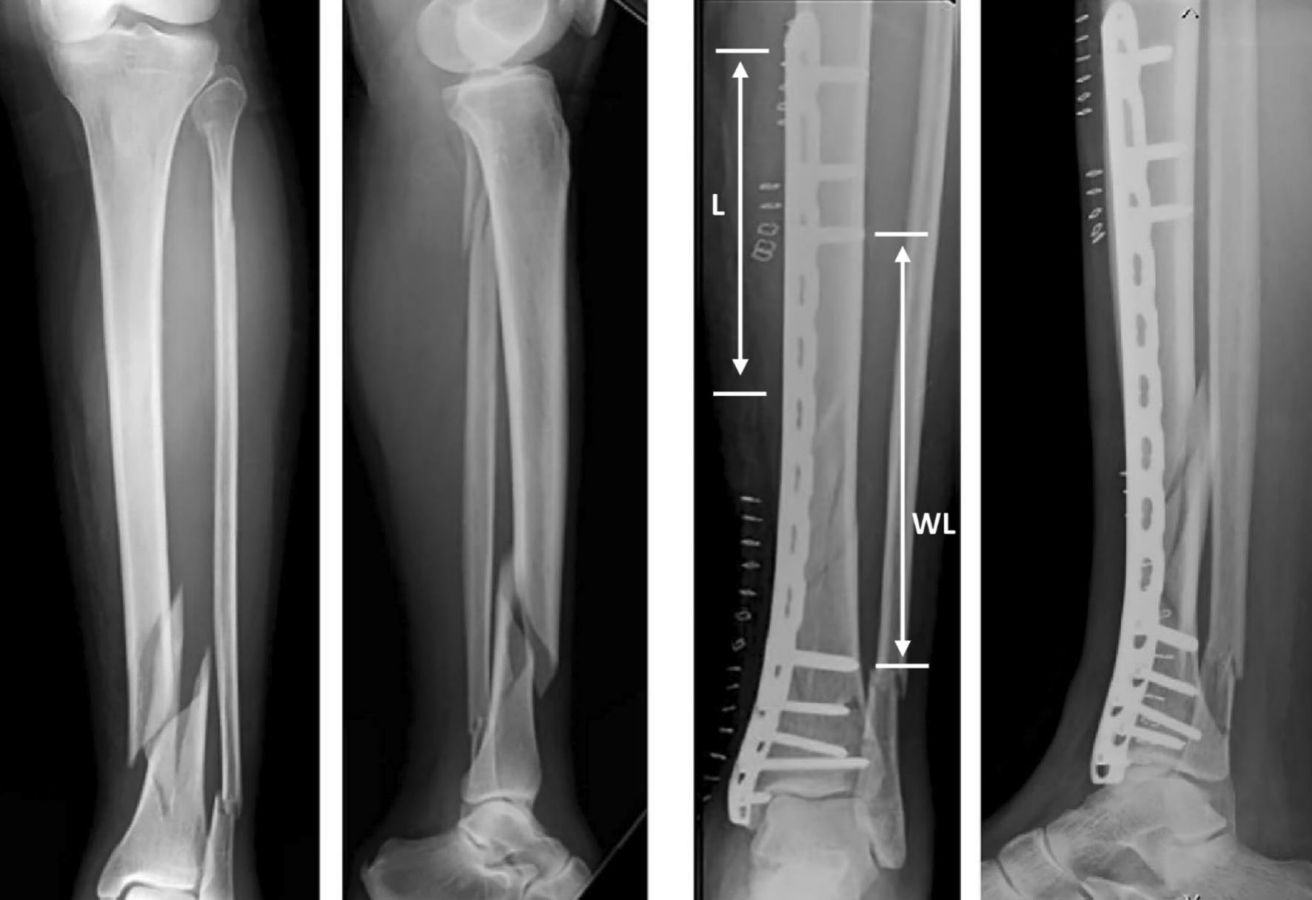
Pre operative radiographs of a left tibia show a 42-B2 fracture with an associated transverse fibula fracture. The tibial fracture was treated with a medial distal tibial bridge plate. Working length is depicted on post operative radiograph (*WL*). Screw density is defined as the length of the plate proximally to the fracture site (*L*) divided by the number of screws proximally. In this radiograph *L* is 112 mm, resulting in a screw density of 37.3 mm

**Fig. 2:**
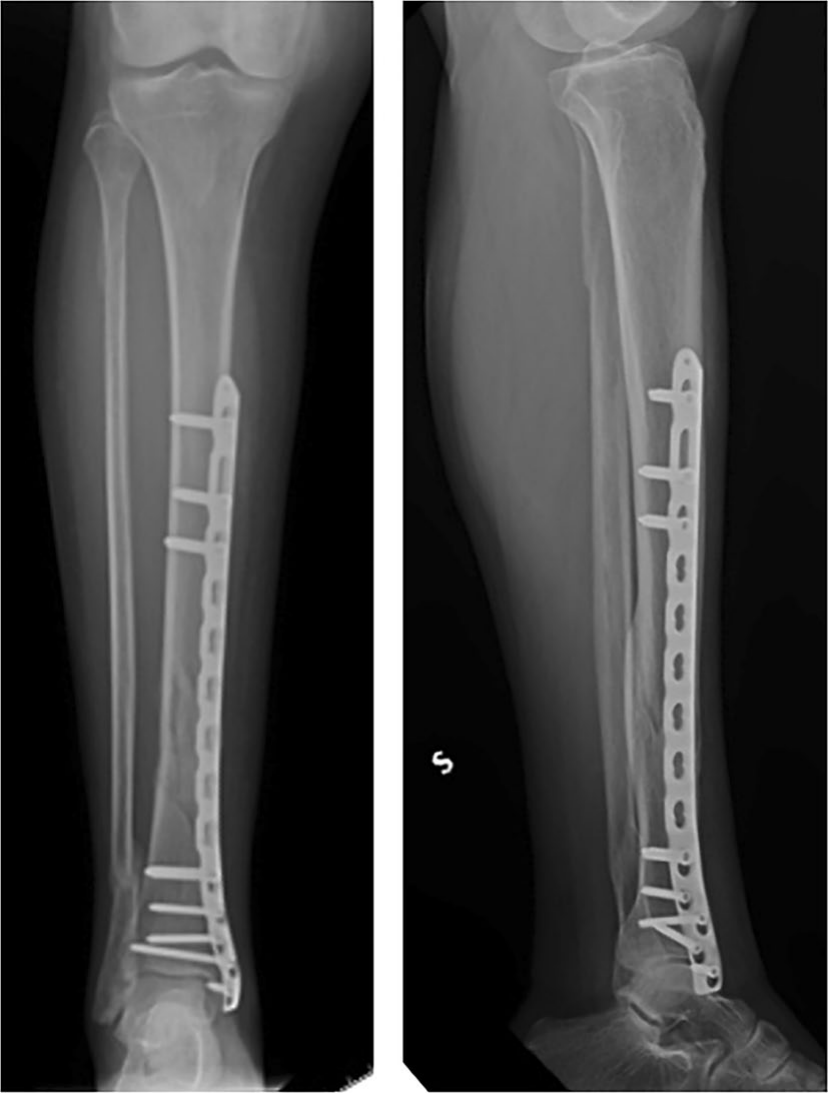
Three months post-operative radiographs show signs of early callus formation with a mRUST score of 10. The fracture went on to heal uneventfully

On post operative radiographs and on operative reports the following parameters were collected: plate length, number and location of the screws, working length (defined as the distance between the two innermost screws in mm) and proximal screw density. According to Harvin et al. proximal screw density was calculated as *L*/*n*, where *L* is the length of the plate proximally to the fracture site and *n* is the number of screws proximally to the fracture. It is expressed in mm [5] (Fig. 1). In distal metaphysis screw placement is dictated by the limited space available and by plate design, thus screw density distally from fracture site wasn’t analyzed. Fracture healing was evaluated according to the modified Radiographic Union Scale in Tibia (mRUST) on AP and LL X-rays 3–6–12 months post-op by two surgeons not involved in the treatment. mRUST defines bone healing based on the number of cortices bridged by callus [5–7]. Union was defined as a minimum mRUST score of 10. Early callus formation was noticed when mRUST score > 8 on 3 months post-surgery radiographs (Fig. 2). In this study the authors analyze whether longer working length and lower screw density were associated with early (< 3 months) callus formation and definitive fracture healing (at 1 year follow-up).

### Statistical analysis

Statistical analysis was performed using SPSS Statistics 20.0 software (IBM, Armonk, NY). Differences in age and sex were analyzed with Pearson’s Chi-squared test and Fisher’s exact test. Differences in fracture type were analyzed with the Freeman–Halton extension of the Fisher’s exact. The Student’s *t* test was performed to compare averages in screw density values and working length values. Association between fibula fixation and callus formation was investigated with Pearson’s Chi-squared test. A *p* value of < 0.05 was considered statistically significant. No power analysis has been performed during the design of the study.

## Results

Forty-two patients met the inclusion criteria: 30 male and 12 female. Mean age was 55.3 year (SD 16.4; 26–85). All fractures were classified according to AO/OTA. A bridge plate osteosynthesis was performed in every patient. LCP Metaphyseal Plate for distal medial tibia 3.5–4.5 was used in every patient. Patients demographic, fracture characteristics and biomechanical parameters are summarized in Table 1.

**Table 1:** Patients demographics and biomechanical parameters in fractures that showed signs of early callus formation (mRUST > 8) and fractures that didn’t

	Callus formation		*p* value
	mRUST>8	mRUST<8	
Sex	22 M; 10 F	8 M; 2 F	0.7
Age (range)	61 (26–92)	54 (39–72)	0.22
> 65 years	19	8	0.29
Fracture type			0.95
42 A	13	5	
42 B	12	3	
42 C	5	1	
43 A	2	1	
Working length (mm)	103.1 (± 32.2)	102.4 (± 37.0)	0.95
Screw density	33.4 (± 5.5)	26.6 (± 9.0)	**0**.**04**

Bold indicates statiscally significant values

Mean working length of the construct was 103.0 mm (SD 32.9; 29.7–168.6). Mean screw density was 31.8 mm (SD 7.0; 17.6–44.0). Working length and screw density values were normally distributed according to Shapiro–Wilk test.

32 patients (76.19%) showed radiographic signs of early callus formation (mRUST score > 8 at 3 months). Patients with signs of early callus were not different to patients without early callus according to age (*p* = 0.29), sex (*p* = 0.70) and fracture type (*p* = 0.95). Mean working length in patients with early callus was 103.1 (± 32.2) mm. In patients who didn’t reach early callus formation the mean working length was 102.4 (± 37.0) mm. No significant difference in the two group was found (*p* = 0.95). Conversely, patients who presented early callus had a significant lower screw density (33.4 mm vs. 26.6 mm, *p* = 0.04).

9 patients (21.4%) had an intact fibula at presentation. Among the 33 fractured fibulas only 20 were fixed surgically (60.6%). 13 patients had a fractured fibula that was not fixed during surgery. Among the patients with a fractured fibula, no difference in early callus formation was noticed if fibula was fixed or left untouched (*p* = 0.39).

39 fractures (92.9%) healed uneventfully, while 3 patients (7.1%) presented major complications, namely one nonunion and two infections, that required further surgical procedures. Of note, none of these patients was smoker or had a diagnosis of diabetes. Two patients healed at 1 year follow-up, one was lost at follow-up. 34 fractures (80.9%) were treated with percutaneous (MIPO) osteosynthesis.

## Discussion

Although distal tibial fractures are very common injuries, some aspects of their treatment remain unclear. In the past two decades many studies have investigated LCP plate osteosynthesis on long bone fractures. Most of them consist of clinical and biomechanical studies focusing on femur [8–12]. Fewer studies focused on the treatment of distal tibia fracture [13, 14]. To the best of our knowledge none investigated how working length and screw density affect construct stability and promote callus formation on distal tibia fractures. These parameters are of paramount importance especially when planning a bridge plate osteosynthesis [15].

Since their introduction locking plates have gained popularity because they showed to provide more stable fixation than conventional non-locking plates, especially in metaphyseal and osteoporotic bone and in highly comminuted fractures. However, some authors argue that LCP plates are too stiff and do not allow the interfragmentary movement needed for callus formation with a rate of insufficient callus formation up to 40% at 6 months follow-up [16].

Increasing the working length is one way to reduce the stiffness of the plate-screw construct [17]. Based on this assumption, we hypothesized that in the context of distal tibia fractures longer working length reduced time to callus formation. Conversely our result showed no significant correlation between callus formation and working length. Our data seems to confirm Parks et al. findings [18]. In their study the authors claim there is no correlation between callus formation and construct stiffness and working length.

We assume that the reason behind the lack of correlation between longer working length and faster callus formation is that in our series many patients (20 out of 42) underwent fibula fracture fixation simultaneously. Our possible explanation is that fixing fibular fracture with a plate may act as a lateral buttress, thus increasing fixation stiffness and preventing valgus collapse of the tibia. This would create a biomechanical environment prone to fracture healing and it would cause the tibial plate to be less dependent on the working length of the construct itself.

Screw density proximally to the fracture site was also evaluated. In our series lower screw density positively correlated with the velocity of callus formation. In patients with an mRUST score > 8 three months post-surgery, a significant lower screw density was noticed. It has been proven that the higher it is the distance between each screw the higher the pull-out force acting on the screws, making the construct less prone to failure and promoting callus formation [4]. However, our results are not aligned with the studies of Harvin et al. and Rodriguez et al. In both these studies the authors found no correlation between screw density and union rates [8, 11]. Nevertheless, a recent study from Jang et al. compared two types of proximal fixation in distal femur plating, namely scattered fixation, in which screws were distant from one another, and clustered fixation, in which screws were spanned over a short segment of the plate. The authors argue that scattering the proximal screws contributed to achieve earlier and more balanced radiographic union in unilateral plating of distal femoral fractures [19].

96.9% of all patients reached uneventful bone healing. Complication rate (nonunions and infections) was 7.1% which is lower than previously reported in literature [20–22]. This can be attributed to the fact open fractures were excluded in our series. Also, many distal tibia fractures undergo a staged treatment at our institution using a temporary external fixator. Finally, in our series the vast majority (34 out of 42) of the patients was treated using minimally invasive approaches and percutaneous application of the implants. MIPO is well known to reduce nonunion risks especially in tibia fractures [23, 24].

Our study has some inherent limitations. First, it is a retrospective study with a limited number of patients. Second, the treatment is not standardized as the two surgeons were free to choose the construct and both simple and comminuted fractures were included. Third, in our series 32 patients reached an mRUST score > 8 at 3 months post-surgery, while only 10 patients didn’t, which is a factor that decreases the power of our statistics. Finally callus formation was assessed on plain film, while a CT scan would have been more accurate. However, unlike in previous clinical studies, we treated our patients with the same tibial plate in titanium, thus avoiding implant selection bias.

## Conclusion

Our findings show that in distal tibia fractures fixed with a medial bridge plate a low screw density is associated with faster callus formation. Conversely, no association was noticed between working length and velocity of callus formation. Our explanation is that in distal tibia fractures—both with intact fibula and where fibula is fixed—the fibula itself acts as a lateral buttress, influencing the biomechanics of the tibial bridge plate and making the construct less dependent on its working length. Future clinical studies with a higher number of patients are needed. Studies comparing screw types (cortical, locking or hybrid) may be necessary. Finally, biomechanical tests and finite element analysis may better clarify the role of working length in distal tibia fractures.
